# Mechanical thrombectomy does not increase the risk of acute symptomatic seizures in patients with an ischaemic stroke: a propensity score matching study

**DOI:** 10.1007/s00415-022-10968-5

**Published:** 2022-01-19

**Authors:** Konstantin Kohlhase, Lisa Marie Tako, Johann Philipp Zöllner, Rejane Golbach, Waltraud Pfeilschifter, Helmuth Steinmetz, Felix Rosenow, Adam Strzelczyk

**Affiliations:** 1 Epilepsy Center Frankfurt Rhine-Main, Department of Neurology, University Hospital Frankfurt, Goethe-University Frankfurt, Frankfurt am Main, Germany; 2 LOEWE Center for Personalized and Translational Epilepsy Research (CePTER), Goethe-University Frankfurt, Frankfurt am Main, Germany; 3 Institute of Biostatistics and Mathematical Modelling, University Hospital Frankfurt, Frankfurt am Main, Germany; 4 Department of Neurology and Neurophysiology, Lüneburg Hospital, Lüneburg, Germany; 5 Center of Neurology and Neurosurgery, University Hospital Frankfurt, Goethe-University Frankfurt, Schleusenweg 2-16, 60528 Frankfurt am Main, Germany

**Keywords:** Epilepsy, Large vessel occlusion, Stroke unit

## Abstract

**Background:**

Mechanical thrombectomy and systemic thrombolysis are important therapies for stroke patients. However, there is disagreement about the accompanying risk of acute symptomatic seizures.

**Methods:**

A retrospective analysis of patients with an acute ischaemic stroke caused by large vessel occlusion was performed. The patients were divided into four groups based on whether they received either mechanical thrombectomy (MT) or systemic thrombolysis (ST; group 1: MT+/ST−; group 2: MT+/ST+; group 3: MT−/ST+; group 4: MT−/ST−). Propensity score matching was conducted for each group combination (1:3, 1:4, 2:3, 2:4, 1:2, 3:4) using the covariates “NIHSS at admission”, “mRS prior to event” and “age”. The primary endpoint was defined as the occurrence of acute symptomatic seizures.

**Results:**

A total of 987 patients met the inclusion criteria, of whom 208, 264, 169 and 346 belonged to groups 1, 2, 3 and 4, respectively. Propensity score matched groups consisted of 160:160, 143:143, 156:156, 144:144, 204:204 and 165:165 patients for the comparisons 1:3, 1:4, 2:3, 2:4, 1:2 and 3:4, respectively. Based on chi-squared tests, there was no significant difference in the frequency of acute symptomatic seizures between the groups. Subgroups varied in their frequency of acute symptomatic seizures, ranging from 2.8 to 3.8%, 2.8–4.4%, 3.6–3.8% and 4.9–6.3% in groups 1, 2, 3 and 4, respectively.

**Conclusion:**

There was no association between MT or ST and an increased risk of acute symptomatic seizures in patients with an acute ischaemic stroke caused by large vessel occlusion who were treated at a primary stroke centre.

**Supplementary Information:**

The online version contains supplementary material available at 10.1007/s00415-022-10968-5.

## Introduction

Strokes are one of the most common causes of death or disability, affecting 1.1 million people per year in Europe alone [1]. Due to demographic change with increase in an aging population, it is expected that this number will continue to increase substantially [1]. In addition, with a probability of up to 50%, strokes are the main cause of epilepsy in individuals over the age of 60 years [2, 3], which is suspected to be associated with a worse overall prognosis for the patient as well as an increased health economic burden [4]. Among patients who experience epileptic seizures, a distinction is made between acute symptomatic seizures within the first 7 days and post-stroke epilepsy beyond 7 days [5]. While acute symptomatic seizures are caused by an increase in cerebral excitability due to an usually reversible disturbance of cerebral homeostasis affecting the blood–brain barrier, ion channel function and neurotransmitter release, post-stroke epilepsy is mostly due to structural changes with chronic inflammation and glioses [6]. In large meta-analyses, the rate of acute symptomatic seizures ranged from 3.3 to 7.0%, while the incidence of post-stroke epilepsy varied between 1.8 and 5.0% [7–9]. Large territorial infarcts, primarily caused by a large vessel occlusion (LVO) of the cerebral arteries, are a risk factor for the occurrence of acute symptomatic seizures, with a positive correlation between scores on the National Institutes of Health Stroke Scale (NIHSS) and the rate of seizures [10–12]. Systemic thrombolysis and mechanical thrombectomy are established procedures to revascularise an occluded vessel and rescue the underlying penumbra from infarction [13]. In particular, mechanical thrombectomy has been proven to achieve a high rate of vessel revascularisation in LVO and an improvement in functional outcome [14]. However, conflicting data exist on whether mechanical recanalisation is associated with an increased rate of acute symptomatic seizures or post-stroke epilepsy due to reperfusion damage [15, 16]. Data from larger registry studies published thus far have not supported this hypothesis [17]. However, most of the studies lacked either a head-to-head comparison with a conservatively treated control group or a matching of patients based on risk factors that are assumed to be linked with acute symptomatic seizures.

In this retrospective single-centre study, we used propensity score matching to evaluate the risk of acute symptomatic seizures in patients treated with mechanical recanalisation compared to patients treated with systemic thrombolysis or conservative treatment in a stroke unit.

## Materials and methods

A retrospective analysis of stroke data from 2016 to 2020 at the University Hospital Frankfurt was performed using a matched case–control design. This analysis was approved by the local ethics committee of the Goethe University Frankfurt. Written informed consent of the patients was waived because the patient data were evaluated retrospectively. Strengthening the Reporting of Observational Studies in Epidemiology (STROBE) guidelines were closely followed [18].

Inclusion criteria were final diagnosis of acute ischaemic stroke according to ICD-10 criteria (International Statistical Classification of Diseases and Related Health Problems, 10th revision) caused by an occlusion of a large cerebral vessel. Ischaemic stroke was defined as a focal neurological deficit that persisted for more than 24 h without appropriate therapy (systemic thrombolysis or mechanical recanalisation) or was accompanied by evidence of irreversible cell damage by additional neuroimaging (MRI or CT). LVO was proven by either vascular imaging (CT or MR angiography) or by infarct demarcation that was only explainable by proximal vessel occlusion (e.g., complete infarction of the middle cerebral artery territory); intracerebral haemorrhage was excluded by the respective neuroimaging. LVO was defined according to the literature as an occlusion of the internal carotid artery, M1 or M2 segment of the middle cerebral artery, A1 or A2 segment of the anterior cerebral artery, P1 or P2 segment of the posterior cerebral artery, the vertebral artery or the basilar artery [19].

The collected data included age; gender; NIHSS at admission, 24 h after admission and at discharge; and modified Rankin Scale (mRS) prior to event and at discharge. In addition, the type of LVO and data from the mechanical thrombectomy, such as the time of vessel revascularisation and the outcome of mechanical thrombectomy according to the Thrombolysis in Cerebral Infarction (TICI) [20] classification, were recorded.

The primary outcome was the occurrence of an acute symptomatic seizure in patients with an acute ischaemic stroke. An acute symptomatic seizure was defined as a clinically apparent epileptic seizure observed by medical personnel or a record of subclinical seizure patterns or nonconvulsive status epilepticus on electroencephalography within the first 7 days after the onset of ischaemic stroke. The onset of stroke was assumed to be either the patient- or third party-reported time of onset or, if this could not be assessed with certainty, the last asymptomatic contact. Time (given as full days) between symptom onset and acute symptomatic seizure was determined. If an acute symptomatic seizure occurred before intervention (mechanical thrombectomy or systemic thrombolysis), this patient was evaluated in the subsequent intervention group.

In addition, semiology of the seizure as well as the changes in electroencephalography (EEG) were assessed. Based on the semiology, the seizures were divided into four groups: (1) seizure with/without impaired awareness but without signs of non-convulsive status epilepticus (NCSE) in EEG, (2) seizure with impaired awareness and proven NCSE in EEG, (3) focal motor seizure, (4) generalized tonic–clonic seizure. The changes in EEG were divided into three groups: (1) no interictal or ictal discharges, (2) interictal discharges, (3) NCSE, (4) no EEG available.

The patient groups were matched using propensity score matching. For this purpose, patients were divided into four groups: patients receiving mechanical recanalisation only (Group 1: MT+/ST−), patients receiving both mechanical recanalisation and systemic thrombolysis (Group 2: MT+/ST+), patients receiving systemic thrombolysis only (Group 3: MT−/ST+) and patients who received neither mechanical recanalisation nor systemic thrombolysis (Group 4: MT−/ST−). The analysis was performed by comparing the respective groups with each other: 1:3, 1:4, 2:3, 2:4, 1:2 and 3:4.

Propensity score matching was performed using the MatchIt package by Ho et al. [21] in RStudio software (RStudio Team [2020]. RStudio: Integrated Development Environment for R. RStudio, PBC, Boston, MA, USA, http://www.rstudio.com/). Matching covariates were “age”, “NIHSS at admission” and “mRS score prior to event”. To reduce the number of unmatched patients, we defined different intervals for the covariates. For age, the intervals were defined as "age" (no interval), "age5" (< 40 years, then in intervals of 5 years), and "age10" (< 40 years, then in intervals of 10). For mRS, we used "mRS1" (no interval) or "mRS2" (mRS 0–1 combined). For NIHSS, "NIHSS1" (no interval), "NIHSS2" (0–3, 4–7, 8–11, 12–15, 16–20, 21–25, 26–30, > 30) and "NIHSS3" (0–3, 4–7, 8–11, 12–15, 16–20, 21–25, > 25) were defined. Matching was accepted if a Wilcoxon test yielded a *p* value > 0.05 for the matching covariates. If more than one matching strategy fulfilled these criteria, the one with the most patients included was used. Final matching was performed with the following covariates: 1:3 ("age10", "NIHSS1", "mRS1"), 1:4 ("age5", "NIHSS2", "mRS1"), 2:3 ("age", "NIHSS1", "mRS2"), 2:4 ("age5", "NIHSS1", "mRS1"), 1:2 ("age", "NIHSS1", "mRS1") and 3:4 ("age", "NIHSS2", "mRS2"). Covariables prior to matching are given in the Supplementary Table 1.

Analysis of descriptive statistics was conducted using SPSS (version 27.0.1.0, IBM Corp., Armonk, NY, USA). Ordinal scaled variables such as NIHSS at admission, NIHSS after 24 h, NIHSS at discharge, mRS prior to event and mRS at discharge were reported using medians (1st–3rd quartiles), whereas age was given as mean ± standard deviation (SD). For statistical testing of the intergroup frequency of acute symptomatic seizures (nominal distribution), a chi-squared test was performed. When comparing patient characteristics between the respective groups, a nonparametric Mann–Whitney *U* test for ordinal and numeric data or a chi-squared test for nominal data was used to determine intergroup differences. Results with a *p* value < 0.05 were determined to be statistically significant. Due to the multiple pairwise comparisons, a Bonferroni correction of *p* value was performed by multiplying it with the number of comparisons (= 6) made per variable.

## Results

**Fig. 1:**
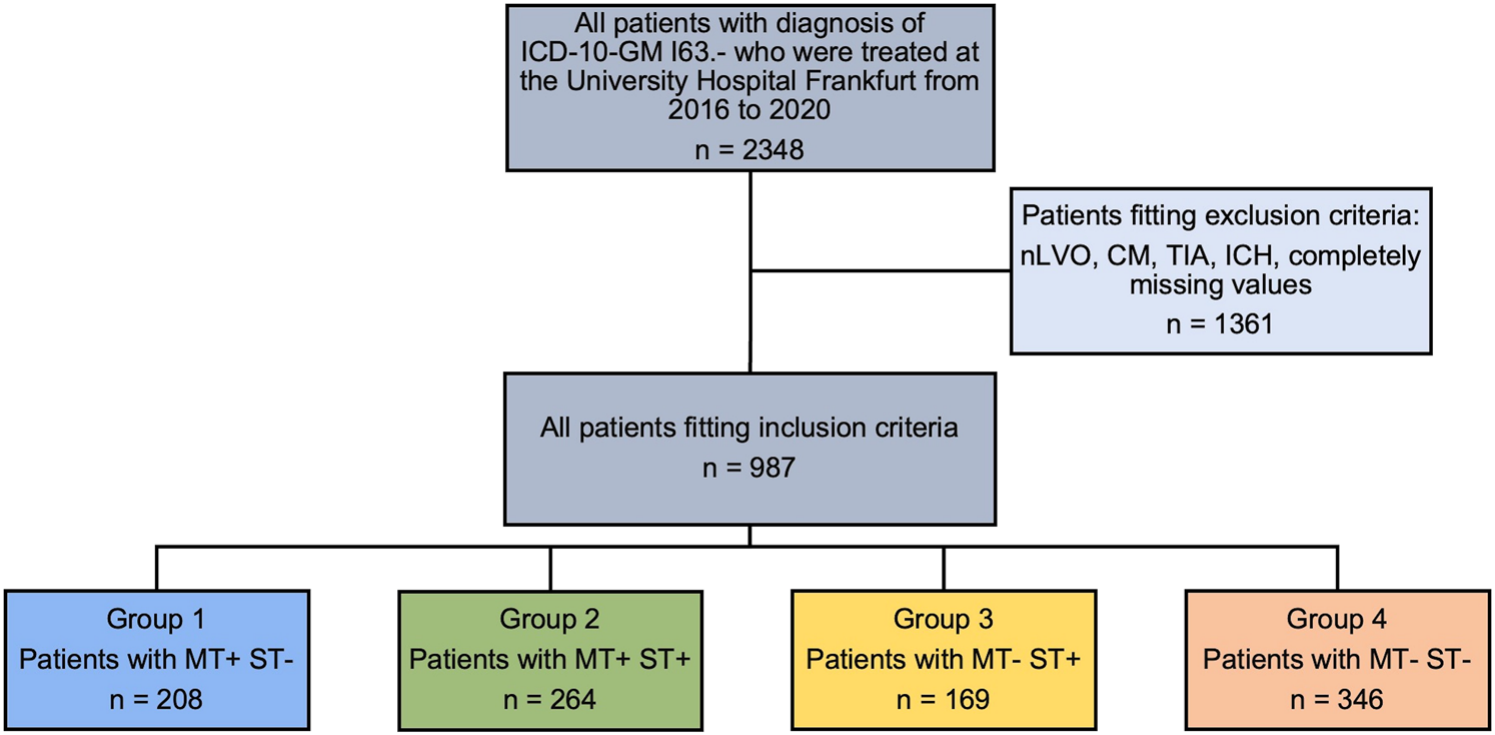
Flowchart showing the patient selection process and the division into four therapy groups. MT−, no mechanical thrombectomy; MT+, mechanical thrombectomy; ST−, no systemic thrombolysis; ST+, systemic thrombolysis; nLVO, non-large vessel occlusion; CM, cerebral microangiopathy; TIA, transient ischaemic attack; ICH, intracerebral haemorrhage

From 2016 to 2020, a total of 987 patients met the inclusion criteria for this study. There were 208, 264, 169 and 346 patients in the MT+/ST−, MT+/ST+, MT−/ST+ and MT−/ST− groups, respectively (Fig. 1). Based on the matching criteria (age, NIHSS at admission and mRS prior to event), the following 1:1 propensity score matchings were calculated: MT+/ST− vs MT−/ST+ (160:160 patients), MT+/ST− vs MT−/ST− (143:143), MT+/ST+ vs MT−/ST+ (156:156), MT+/ST+ vs MT−/ST− (144:144), MT+/ST− vs MT+/ST+ (204:204) and MT−/ST+ vs MT−/ST− (165:165; Fig. 2).

**Fig. 2:**
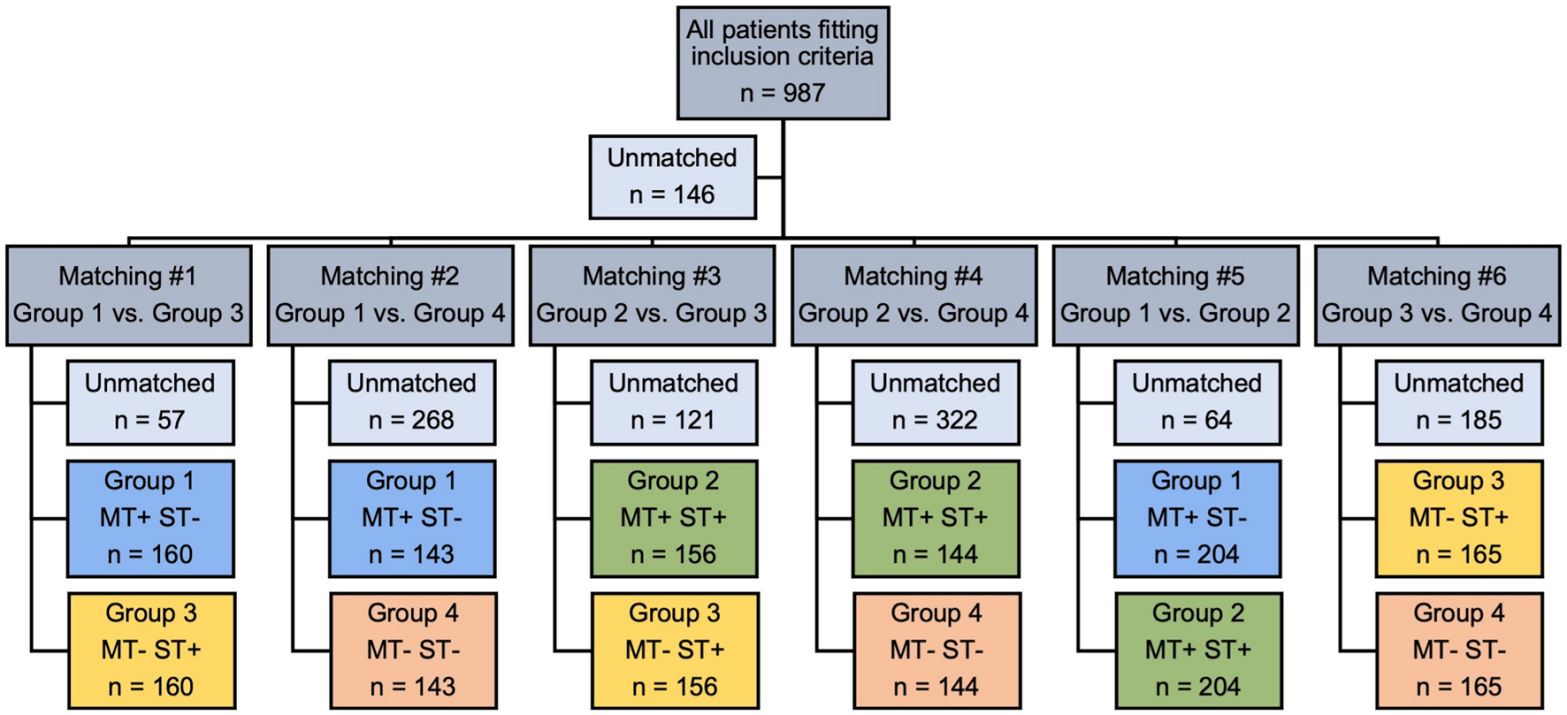
Flowchart showing the number of patients in the different matchings of therapy groups. ST−, no systemic thrombolysis; ST+, systemic thrombolysis; MT−, no mechanical thrombectomy; MT+, mechanical thrombectomy

After correction for multiple testing, no significant differences in the defined parameters (age, NIHSS at admission, mRS prior to event) were observed between the matched groups (Table 1). Only Matching 1:3 showed significant differences in NIHSS after 24h and NIHSS at discharge, while Matching 2:4 was significant different in mRS at discharge. There was also no significantly higher mortality rate during hospital stay, however there was a trend towards higher mortality rates in the MT−/ST− group compared with the MT+/ST+ and MT+/ST− groups, as well as a higher mortality rate in the MT−/ST+ group compared with the MT+/ST− group. The latency from symptom onset to mechanical thrombectomy was shorter and the outcome after mechanical thrombectomy (TICI) was better in patients who received systemic thrombolysis (MT+/ST+) than in those who did not (MT+/ST−). Regarding the distribution of vessel occlusions, significantly more M1 occlusions were observed in the mechanical recanalisation groups (MT+/ST− and MT+/ST+) compared to both groups without mechanical recanalisation (MT−/ST+ and MT−/ST−). In contrast, a significant difference in internal carotid artery occlusion was not observed between these groups. Regarding the posterior circulation, an occlusion of the P1 or P2 segment of the posterior cerebral artery was numerically more frequent in the groups without mechanical recanalisation (MT−/ST− or MT−/ST+) compared to the groups who underwent mechanical recanalisation (MT+/ST+ or MT+/ST−) without reaching significance. Furthermore, there were significantly more vertebral artery occlusions in patients who did not receive any therapy (MT−/ST−) compared to patients who received mechanical thrombectomy with systemic thrombolysis (MT+/ST+). For further details, please refer to Table 1.

**Table 1:** Patient characteristics among the respective matching groups

	MT+/ST-	MT-/ST+	p	MT+/ST-	MT-/ST-	*p*	MT+/ST+	MT-/ST+	*p*	MT+/ST+	MT-/ST-	*p*	MT+/ST-	MT+/ST+	*p*	MT-/ST+	MT-/ST-	*p*
*n*	160	160	–	143	143	–	156	156	–	144	144	–	204	204	–	165	165	–
Age, mean ± SD	73 ± 13	75 ± 13	0.6	69 ± 15	72 ± 14	0.42	73 ± 11	75 ± 13	0.42	69 ± 13	71 ± 15	0.66	70 ± 14	69 ± 14	1.0	75 ± 13	76 ± 12	1.0
Gender %Female	52.5%	51.9%	1.0	48.3%	46.9%	1.0	54.5%	51.3%	1.0	45.8%	47.9%	1.0	48%	49%	1.0	52.1%	50.9%	1.0
NIHSS, median (*Q*1–*Q*3), *n*=missing data																		
On admission	13 (9–17)	11 (4–17)	0.42	13 (8–18)	14 (8–19)	1.0	12 (8–16)	10 (4–17)	1.0	13 (6–18)	12 (6–17)	1.0	14 (10–18)	14 (9–18)	1.0	10 (4–17)	11 (4–18)	1.0
After 24h	9 (4–16), n=38	6 (1–16), n=41	**0.012**	9 (4–18), n=38	11 (5–18), n=56	1.0	7 (2–13), n=49	5 (1–14), n=39	1.0	6 (2–14), n=35	9 (4–17), n=47	0.06	10 (4–17), n=53	7 (3–15), n=57	0.06	5 (1–15), n=41	6 (2–16), n=49	0.66
At discharge	5 (1–11), n=56	2 (0–6), n=59	**0.006**	4 (1–11), n=51	6 (2–13), n=71	1.0	2 (0–6), n=59	2 (0–6), n=55	1.0	3 (0–7), n=48	5 (2–12), n=58	0.12	5 (1–11), n=70	3 (1–10), n=73	1.0	2 (0–6), n=105	3 (1–12), n=63	0.06
mRS, median (*Q*1–*Q*3), *n*=missing data																		
Prior to admission	0 (0–2)	1 (0–2)	1.0	0 (0–2)	1 (0–2)	0.36	0 (0–1)	0 (0–2)	1.0	1 (0–1)	1 (0–1)	1.0	0 (0–1)	0 (0–1)	1.0	1 (0–2)	1 (0–2)	1.0
At discharge	4 (2–5), *n*=26	4 (1–6), *n*=21	1.0	4 (2–5), *n*=24	4 (3–6), *n*=21	0.18	3 (1–5), *n*=26	3 (1–6), *n*=19	1.0	3 (1–5), *n*=19	4 (2–6), *n*=16	**0.012**	4 (2–5), *n*=34	4 (1–5), *n*=35	1.0	4 (1–6), *n*=21	4 (2–6), *n*=18	0.48
Acute symptomatic seizures	6, 3.8%	6, 3.8%	1.0	4, 2.8%	9, 6.3%	1.0	5, 3.2%	6, 3.8%	1.0	4, 2.8%	7, 4.9%	1.0	7, 3.4%	9, 4.4%	1.0	6, 3.6%	9, 5.5%	1.0
Died during stay	15%	23.8%	0.5	15.4%	28.7%	0.06	17.9%	23.1%	1.0	14.6%	25.7%	0.12	14.7%	14.2%	1.0	23%	24.2%	1.0
Occlusion																		
M1	97, 60.6%	52, 32.5%	**0.006**	76, 53.1%	46, 32.2%	**<0.01**	88, 56.4%	52, 33.3%	**<0.01**	80, 55.6%	39, 27.1%	**<0.01**	117, 57,4%	116, 56.9%	1.0	55, 33.3%	48, 29.1%	1.0
M2	35, 21.9%	52, 32.5%	0.18	29, 20.3%	30, 21.0%	1.0	35, 22.4%	49, 31.4%	0.42	35, 24.3%	39, 27.1%	1.0	41, 20.1%	41, 20.1%	1.0	53, 32.1%	35, 21.2%	0.18
ICA	41, 25.6%	35, 21.9%	1.0	37, 25.9%	51, 35.7%	0.42	45, 28.8%	35, 22.4%	1.0	40, 27.8%	50, 34.7%	1.0	57, 27.9%	64, 31.4%	1.0	35, 21.2%	54, 32.7%	0.12
A1	2, 1.3%	0, 0.0%	1.0	1, 0.7%	0, 0.0%	1.0	0, 0.0%	0, 0.0%	1.0	0, 0.0%	0, 0.0%	1.0	2, 1.0%	0, 0.0%	1.0	0, 0.0%	1, 0.6%	1.0
A2	1, 0.6%	2, 1.3%	1.0	0, 0.0%	1, 0.7%	1.0	0, 0.0%	1, 0.6%	1.0	1, 0.7%	1, 0.7%	1.0	2, 1.0%	1, 0.5%	1.0	2, 1.2%	2, 1.2%	1.0
P1	2, 1.3%	3, 1.9%	1.0	2, 1.4%	4, 2.8%	1.0	1, 0.6%	3, 1.9%	1.0	2, 1.4%	9, 6.3%	0.18	2, 1.0%	2, 1.0%	1.0	3, 1.8%	6, 3.6%	1.0
P2	0, 0.0%	5, 3.1%	0,12	0, 0.0%	5, 3.5%	0.12	1, 0.6%	5, 3.2%	0.6	2, 1.4%	6, 4.2%	0.9	0, 0.0%	2, 1.0%	1.0	5, 3.0%	5, 3.0%	1.0
VA	4, 2.5%	8, 5.0%	1.0	5, 3.5%	14, 9.8%	0.18	2, 1.3%	8, 5.1%	1.0	2, 1.4%	13, 9.0%	**0.024**	6, 2.9%	3, 1.5%	1.0	8, 4.8%	21, 12.7%	0.06
BA	13, 8.1%	12, 7.5%	1.0	18, 12.6%	14, 9.8%	1.0	15, 9.6%	11, 7.1%	1.0	14, 9.7%	11, 7.6%	1.0	20, 9.8%	21, 10.3%	1.0	12, 7.3%	13, 7.9%	1.0
Latency from symptom onset to mechanical thrombectomy																		
Unknown	72, 45%	–	–	75, 52.4%	–	–	37, 23.7%	–	–	38, 26.4%	–	–	99, 48.5%	50, 24.5%	**0.001**	–	–	–
<6 h	63, 39.4%	–	–	47, 32.9%	–	–	104, 66.7%	–	–	95, 66.0%	–	–	78, 38.2%	140, 68.6%		–	–	–
6–16 h	23, 14.4%	–	–	19, 13.3%	–	–	15, 9.6%	–	–	11, 7.6%	–	–	25, 12.3%	14, 6.9%		–	–	–
16–24 h	1, 0.6%	–	–	1, 0.7%	–	–	0, 0.0%	–	–	0, 0.0%	–	–	1, 0.5%	14, 6.9%		–	–	–
Outcome (TICI)																		
2b, III	130, 81.3%	–	–	116, 81.1%	–	–	140, 89.7%	–	–	134, 93.1%	–	–	161, 78,9%	185, 90.7%	**< 0.01**	–	–	–
< 2b	30, 18.8%	–	–	27, 18.9%	–	–	16, 10.3%	–	–	10, 6.9%	–	–	43, 21,1%	19, 9.3%		–	–	–

Intergroup differences were measured using Mann-Whitney U test for ordinal and numeric values (age, NIHSS at admission, NIHSS after 24 hours, NIHSS at discharge, mRS prior to admission and mRS at discharge), whereas chi-squared test was used for nominal values (gender, type of vessel occlusion). Bonferroni correction of *p* values was conducted for multiple pairwise testing by multiplying it with the number of comparisons made in each variable (*n* = 6). A *p* value < 0.05 was determined significant. (Abbreviations: *SD* standard deviation, *Q1* first quartile, *Q3* third quartile, *mRS* Modified Rankin Scale, *MT* mechanical thrombectomy, *ST* systemic thrombolysis, *TICI* Thrombolysis in Cerebral Infarction, *M1 and M2* M1 and M2 segment of middle cerebral artery, *ICA* internal carotid artery, *A1 and A2* A1 and A2 segment of anterior cerebral artery, *P1 and P2* P1 and P2 segment of posterior cerebral artery, *VA* vertebral artery, *BA* basilar artery)

**Fig. 3:**
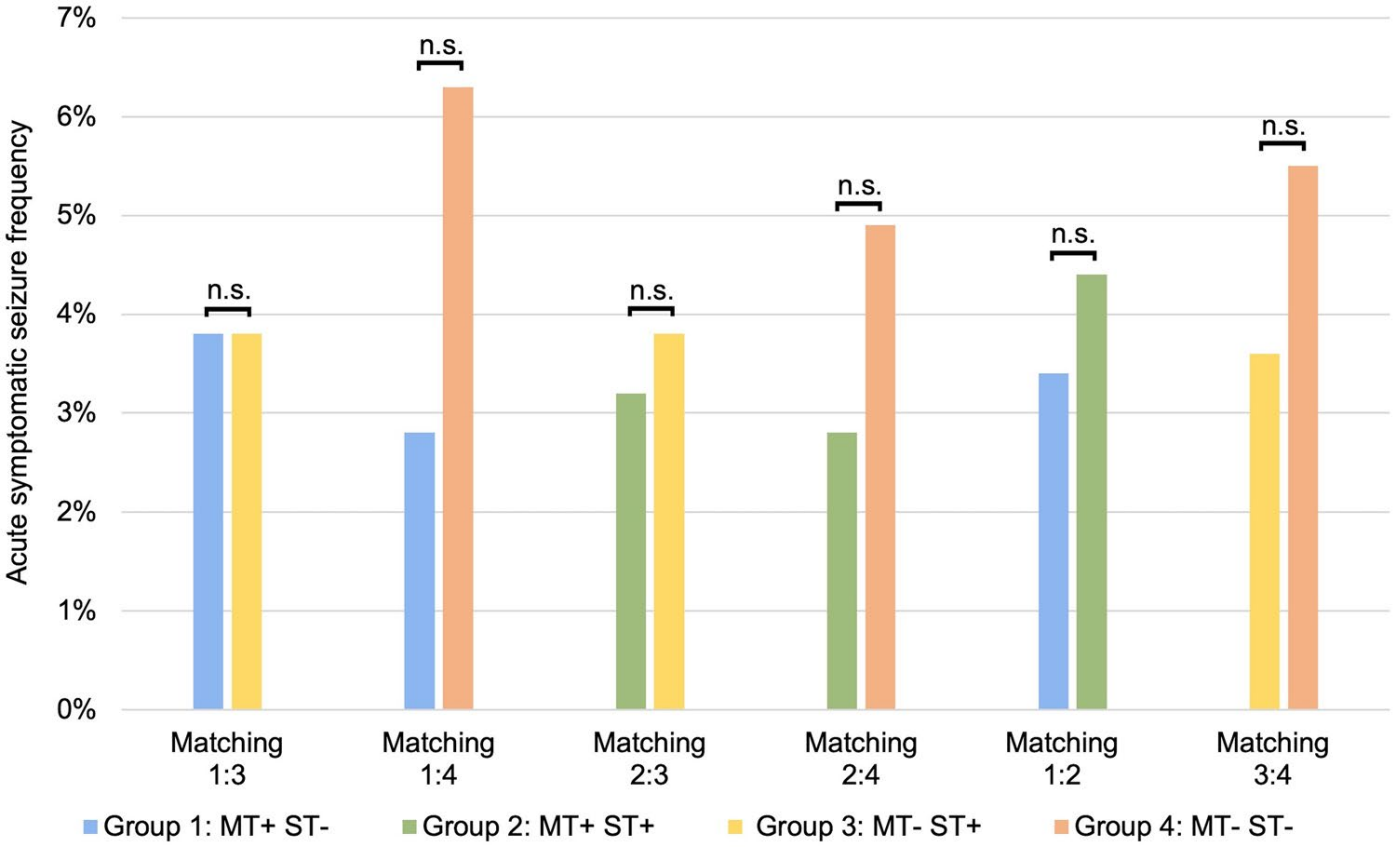
Acute symptomatic seizure frequency in different treatment groups. ST−, no systemic thrombolysis; ST+, systemic thrombolysis; MT−, no mechanical thrombectomy; MT+, mechanical thrombectomy (chi-squared tests: not significant)

The frequency of acute symptomatic seizures was not significantly different between the matched groups: MT+/ST− vs MT−/ST+, Phi = 0.0, *p* = 1.0, p* (* = corrected for multiple testing) = 1.0; MT+/ST− vs MT−/ST−, Phi = 0.08, *p* = 0.156, p* = 1.0; MT+/ST+ vs MT−/ST+, Phi = 0.02, *p* = 0.76, p* = 1.0; MT+/ST+ vs MT−/ST−, Phi = 0.05, *p* = 0.36, p* = 1.0; MT+/ST− vs MT+/ST+, Phi = 0.025, *p* = 0.61, p* = 1.0; MT−/ST+ vs MT−/ST−, Phi = 0.04, *p* = 0.428, p* = 1.0. The frequency of acute symptomatic seizures among the groups created by each matching varied, ranging between 2.8 and 3.8% after mechanical recanalisation without systemic thrombolysis (MT+/ST−, group 1) and between 2.8 and 4.4% after mechanical recanalisation with systemic thrombolysis (MT+/ST+, group 2). In patients without mechanical recanalisation but with systemic thrombolysis (MT−/ST+, group 3), the risk of acute symptomatic seizures was 3.6–3.8%, whereas without mechanical thrombectomy or thrombolysis (MT−/ST−, group 4), the risk was 4.9–6.3% (Table 1 and Fig. 3).

Acute symptomatic seizures with impaired awareness without NCSE (1) and with NCSE (2) were found in 1.6% and 0.5%, 1.0% and 2.4%, 0.6% and 0.0%, as well as 2.2% and 0.4% in groups 1, 2, 3, and 4, respectively. Focal motor seizure (3) was reported in 0.0%, 0.5%, 1.2%, and 1.3%, whereas GTCS (4) was found in 1.0%, 0.0%, 1.8%, and 1.3% in groups 1, 2, 3, and 4, respectively. In subgroup analysis of patients with GTCS, chi-square test did not show significant differences between the respective groups. Among patients with acute symptomatic seizures, no interictal discharges (1) were found in 33.3% (2/6 = results of EEG/number of acute symptomatic seizures within group), 12.5% (1/8), 66.6% (4/6) and 50% (6/12) in patients of group 1, 2, 3 and 4, respectively. Interictal discharges (2) or NCSE (3) were found in 50% (3/6) and 16.6% (1/6), 12.5% (1/8) and 62.5% (5/8), 33.3% (2/6) and 0.0% (0/6), and 25% (3/12) and 8.3% (1/12) in groups 1, 2, 3, and 4, respectively (Table 2). No EEG was available in a total of three patients (Group 2 = 1 patient, Group 4 = 2 patients). Seizures occurred at a median (minimum–maximum) of 3 (1–7), 3 (0–7), 0 (0–3), and 2 (0–7) days after symptom onset in patients of group 1, 2, 3, and 4, respectively.

**Table 2:** Semiology and results of electroencephalography (EEG) in patients with acute symptomatic seizures, (*NCSE* non-convulsive status epilepticus, *GTCS* generalized tonic–clonic seizure), where EEG results are given as a ratio to the number of acute symptomatic seizures among the respective group

	MT+/ST− (*n* = 191)	MT+/ST+ (*n* = 208)	MT−/ST+ (*n* = 165)	MT−/ST− (*n* = 229)
Semiology				
Impaired awareness without NCSE in EEG	1.6% (*n* = 3)	1.0% (*n* = 2)	0.6% (*n* = 1)	2.2% (*n* = 5)
Impaired awareness with NCSE in EEG	0.5 (*n* = 1)	2.4% (*n* = 5)	0.0% (*n* = 0)	0.4% (*n* = 1)
Focal motor seizure	0.0% (*n* = 0)	0.5% (*n* = 1)	1.2% (*n* = 2)	1.3% (*n* = 3)
GTCS	1.0% (*n* = 2)	0.0% (*n* = 0)	1.8% (*n* = 3)	1.3% (*n* = 3)
EEG				
No discharges	33.3% (*n* = 2/6)	12.5% (*n* = 1/8)	66.6% (*n* = 4/6)	50% (*n* = 6/12)
Interictal discharges	50.0% (*n* = 3/6)	12.5% (*n* = 1/8)	33.3% (*n* = 2/6)	25.0% (*n* = 3/12)
NCSE	16.6% (*n* = 1/6)	62.5% (*n* = 5/8)	0.0% (*n* = 0)	8.3% (*n* = 1/12)
No EEG	0.0% (*n* = 0)	12.5% (*n* = 1/8)	0.0% (*n* = 0)	16.7% (*n* = 2/12)

Using chi-squared test, there was no significant difference in GTCS between the groups

## Discussion

Mechanical thrombectomy and systemic thrombolysis are the fundamental treatment tools for stroke care in patients with LVO, and the aim of their use is to minimise the extent of irreversible brain damage. However, recent studies have suggested that emergent reopening therapy using systemic thrombolysis or mechanical recanalisation might carry an increased risk of acute symptomatic seizures.

In this study, we demonstrated that revascularisation of LVO with mechanical thrombectomy, intravenous thrombolysis or a combination of these therapies was not associated with an increased risk of acute symptomatic seizures. These results are consistent with the recently published findings of Zöllner et al., who also found no increased risk of acute symptomatic seizures after mechanical thrombectomy or systemic thrombolysis in a retrospective analysis of the Quality Assurance Office Hessen Stroke Registry including a total of 135,117 patients [17]. Since the latter study was a population-based register study with a high number of cases from different centres, it carried the potential disadvantage of heterogeneous quality of reported clinical data, such as the evaluation of acute symptomatic seizures [22] or incomplete collection of data regarding the underlying vascular occlusion [23]. In contrast, in our patient-centred evaluation of stroke data from a primary university stroke centre, only patients with a causative LVO were included and propensity score matched based on relevant criteria (age, NIHSS and mRS), thus reducing the risk of bias in determining the influence of mechanical recanalisation or systemic thrombolysis on the occurrence of acute symptomatic seizures.

However, some studies have suggested an increased risk of acute symptomatic seizures after systemic thrombolysis [24]. Neurotoxicity of thrombolysis was suspected as a possible cause [25]. For example, Alvarez et al. reported a significantly increased risk of acute symptomatic seizures among thrombolysed patients, with an OR of 4.6, although the small number of thrombolysed (both systemic and intra-arterial) patients (*n* = 11) and the fact that the control group was randomly selected rather than matched limited the statistical robustness of the results [24]. Furthermore, there was a significantly higher NIHSS score at admission (14.8 vs 9.35) between the thrombolysed patients compared with the control group; notably, a higher NIHSS score is more likely associated with a higher stroke volume and positively correlates with the risk of acute symptomatic seizures [3, 11, 26, 27]. Similarly, De Reuck et al. also reported an increased risk of acute symptomatic seizures after systemic thrombolysis, but attributed this less to a direct effect of thrombolysis than to the severity of the stroke [28]. By including only patients with an LVO in all groups in our study and matching them according to the NIHSS score, we minimised the influence of this selection bias.

Regarding post-stroke epilepsy within 2 years, which is pathophysiologically distinct from acute symptomatic seizures, Naylor et al. showed an increased risk in patients with anterior circulation cerebral infarction and systemic thrombolysis or mechanical recanalisation (adjusted OR 3.4–5.5) [15]. However, there was no significant difference between the therapy groups; thus, the reperfusion itself rather than the procedure was assumed to be the actual cause. Pathophysiologically, it was suspected that reperfusion may lead to a disturbance of the blood–brain barrier, with release of free radicals and increased cerebral excitability, resulting in an increased risk of acute symptomatic seizures [29]. In contrast, our results revealed the highest rate of acute symptomatic seizures in the group that did not receive any therapy (MT−/ST−), although a statistically significant difference was not reached. With a reperfusion rate of 78.9–93.1%, the majority of patients receiving mechanical thrombectomy had a successful intervention and thus might bear an increased risk of reperfusion syndrome. Since the risk of reperfusion syndrome depends largely on hypertensive blood pressure during the acute phase after stroke [30], all included patients with acute stroke were treated on a stroke unit to facilitate regular blood pressure monitoring. Blood pressure was intensively controlled for up to 72 h, with a targeted value of < 140 mmHg in case of successful vessel reopening or 140–160 mmHg in case of persistent vessel occlusion after systemic thrombolysis or mechanical recanalisation. With these measures in place, we did not observe an increased risk of acute symptomatic seizures after mechanical recanalisation in a controlled setting on a stroke unit.

There were some significant differences between the treatment groups regarding the location of the LVO. In particular, the groups of patients who received mechanical thrombectomy had a significantly higher proportion of M1 occlusions compared with the MT−/ST+ or MT−/ST− groups. This is due to the fact that M1 occlusion is mostly associated with a severe neurological deficit and a large penumbra volume [31]. In addition, the therapeutic benefit of MT has been validated predominantly for patients with a proximal LVO of the anterior circulation, especially an M1 occlusion [11, 32, 33]. Although there was no statistically significant difference in acute symptomatic seizures between any of the groups, the highest rate of acute symptomatic seizures was observed in patients who did not receive a reperfusion treatment (MT−/ST−), who typically showed the largest final infarct volume, which is a risk factor for the occurrence of acute symptomatic seizures [11].

This study had several limitations. Although this study evaluated one of the largest monocentric cohorts so far, the limited number of acute symptomatic seizures might increase the risk of overlooking a potential difference between the groups. Furthermore, matching regarding the underlying vessel occlusion or success of recanalisation was not possible due to the limited number of available cases. The final infarct size was approximated by stroke severity recorded by NIHSS and not radiologically determined. Studies that include larger number of patients and therefore allow for a matching based on the final infarct size and vessel occlusion would be necessary. In addition, systematic comparison between hospitals with and without stroke unit care would allow to assess the impact of consistent prevention of reperfusion syndrome. Propensity score matching has been described as a method for quasi-randomization of observational studies, however it bears the risk of underestimating latent covariates since matching was only based on measured variables such as age, NIHSS and mRS. An example of a latent variable would be cerebral seizure propensity and the extent of cortical damage. The use of NIHSS can mitigate this to some extent. Other variables such as infarct volume on cerebral imaging or hemorrhagic transformation can be included and improve this estimate and can incorporate the effect of latent variables into the statistical analysis through statistical models [33]. Because adjustment for latent variables was not performed in our study, this should be considered in the assessment of the results.

## Conclusion

We did not identify an association between mechanical thrombectomy or systemic thrombolysis and an increased risk of acute symptomatic seizures in patients with an acute stroke resulting from an LVO who were treated at a primary stroke centre.

## Supplementary Information

Below is the link to the electronic supplementary material.

Supplementary file1 (DOCX 15 KB)
