# Correction to: Mechanical thrombectomy does not increase the risk of acute symptomatic seizures in patients with an ischaemic stroke: a propensity score matching study

**DOI:** 10.1007/s00415-022-11062-6

**Published:** 2022-04-08

**Authors:** Konstantin Kohlhase, Lisa Marie Tako, Johann Philipp Zöllner, Rejane Golbach, Waltraud Pfeilschifter, Helmuth Steinmetz, Felix Rosenow, Adam Strzelczyk

**Affiliations:** 1 Epilepsy Center Frankfurt Rhine-Main, Department of Neurology, University Hospital Frankfurt, Goethe-University Frankfurt, Frankfurt am Main, Germany; 2 LOEWE Center for Personalized and Translational Epilepsy Research (CePTER), Goethe-University Frankfurt, Frankfurt am Main, Germany; 3 Institute of Biostatistics and Mathematical Modelling, University Hospital Frankfurt, Frankfurt am Main, Germany; 4 Department of Neurology and Neurophysiology, Lüneburg Hospital, Lüneburg, Germany; 5 Center of Neurology and Neurosurgery, University Hospital Frankfurt, Goethe-University Frankfurt, Schleusenweg 2-16, 60528 Frankfurt am Main, Germany

## Correction to: Journal of Neurology 10.1007/s00415-022-10968-5

The original version of this article unfortunately contained errors in the formatting of Table 1.

Table 1 was corrected: The formatting of Table 1 has been adjusted. Values that were erroneously marked in bold have been corrected. The abbreviation ACI was changed to ICA, AV to VA and AB to BA. The data in column “MT-/ST+” in line NIHSS - “On admission” should read as 11 (4–17) instead of 11 (4.25–17). In addition, the stated mRS in column “MT-/ST+” in row “At discharge” was corrected from 1 (1–6) to 4 (1–6).

The original article has been corrected.

