# Supplementary material for: Mechanical thrombectomy does not increase the risk of acute symptomatic seizures in patients with an ischaemic stroke: a propensity score matching study
Source: J Neurol. 2022 Jan 19;269(6):3328–36. doi: 10.1007/s00415-022-10968-5 (PMC9119889; doi:10.1007/s00415-022-10968-5)
Supplement: Supplementary file 1 — Supplementary file1 (DOCX 15 KB) [file 415_2022_10968_MOESM1_ESM.docx]

| Supplementary Table 1 | MT+/ST-  (Group I) | MT+/ST+ (Group 2) | MT-/ST+ (Group 3) | MT-/ST-  (Group 4) |
| --- | --- | --- | --- | --- |
| Number of patients | 208 | 264 | 169 | 346 |
| Age, years  (mean ± standard deviation) | 70 ± 14 | 69 ± 14 | 75 ± 13 | 71 ± 14 |
| NIHSS on admission | 14 (10 - 18) | 14 (9 – 18) | 10 (4 – 17) | 6 (2 – 16) |
| mRS prior to admission | 0 (0 - 2) | 0 (0 – 1) | 1 (0 – 2) | 1 (0 – 2) |

Supplementary Table 1: Patient characteristics prior to matching. NIHSS on admission and mRS prior to admission is given as median (interquartile range). (Abbreviations: MT = mechanical thrombectomy, ST = systemic thrombolysis, NIHSS = National Institute of Health Stroke Scale, mRS = Modified Rankin Scale)
